# A buffered media system for yeast batch culture growth

**DOI:** 10.1186/s12866-021-02191-5

**Published:** 2021-04-23

**Authors:** Rianne C. Prins, Sonja Billerbeck

**Affiliations:** Molecular Microbiology, Groningen Biomolecular Sciences and Biotechnology Institute, University of Groningen, Groningen, The Netherlands

**Keywords:** Yeast, Growth medium, Buffer, pH, Secreted proteins

## Abstract

**Background:**

Fungi are premier hosts for the high-yield secretion of proteins for biomedical and industrial applications. The stability and activity of these secreted proteins is often dependent on the culture pH. As yeast acidifies the commonly used synthetic complete drop-out (SD) media that contains ammonium sulfate, the pH of the media needs to be buffered in order to maintain a desired extracellular pH during biomass production. At the same time, many buffering agents affect growth at the concentrations needed to support a stable pH. Although the standard for biotechnological research and development is shaken batch cultures or microtiter plate cultures that cannot be easily automatically pH-adjusted during growth, there is no comparative study that evaluates the buffering capacity and growth effects of different media types across pH-values in order to develop a pH-stable batch culture system.

**Results:**

We systematically test the buffering capacity and growth effects of a citrate-phosphate buffer (CPB) from acidic to neutral pH across different media types. These media types differ in their nitrogen source (ammonium sulfate, urea or both). We find that the widely used synthetic drop-out media that uses ammonium sulfate as nitrogen source can only be effectively buffered at buffer concentrations that also affect growth. At lower concentrations, yeast biomass production still acidifies the media. When replacing the ammonium sulfate with urea, the media alkalizes. We then develop a medium combining ammonium sulfate and urea which can be buffered at low CPB concentrations that do not affect growth. In addition, we show that a buffer based on Tris/HCl is not effective in maintaining any of our media types at neutral pH even at relatively high concentrations.

**Conclusion:**

Here we show that the buffering of yeast batch cultures is not straight-forward and addition of a buffering agent to set a desired starting pH does not guarantee pH-maintenance during growth. In response, we present a buffered media system based on an ammonium sulfate/urea medium that enables relatively stable pH-maintenance across a wide pH-range without affecting growth. This buffering system is useful for protein-secretion-screenings, antifungal activity assays, as well as for other pH-dependent basic biology or biotechnology projects.

**Supplementary Information:**

The online version contains supplementary material available at 10.1186/s12866-021-02191-5.

## Background

The extracellular pH is an important environmental factor, both for the functioning of fungi in their natural habitats as well as for engineered functionalities of fungi in the shake flask of a biotechnologist.

Unicellular fungi such as the yeasts *Saccharomyces cerevisiae* or *Kluyveromyces lactis* are widely used cell factories for the secretion of recombinant proteins [1, 2]. Many secreted proteins require a specific culture pH to stay functional. For example, antifungal proteins (so-called yeast killer toxins) often require a low pH to be functional [3]. Other proteins, such as α-galactosidase, have shown to require a neutral culture pH and lose activity in media that acidifies during biomass production [4]. Further, the stability and potency of many small molecule antifungals is pH dependent [5, 6].

As such, protein pH activity profiling, antifungal testing and determination of minimal inhibitory concentrations should ideally be performed at a defined pH.

In a similar way, the action of yeast antibiotics that are commonly used for selection purposes in genetic engineering (e.g. G418 or Nourseothricin) are also dependent on a neutral culture pH for functionality [7].

pH is furthermore an important environmental trigger, influencing metabolism [8, 9], life-span [10], metabolite production performance [11], biofilm formation and virulence [12–15] of various biotechnologically or medically relevant yeast, and thus important when performing fundamental science experiments as well.

Growth in shaken batch cultures, microtiter plates or on solid agar plates are the leading experimental set-ups for basic fungal biology studies or biotechnological developments. These set-ups do not allow for automated pH maintenance and the media needs to be buffered to maintain a desired culture pH during biomass production. Using a reliable buffering system provided in sufficient concentration is important as biomass production leads to strong acidification of the most commonly used synthetic drop-out media that employs ammonium sulfate as nitrogen source [4]. At the same time we observed in our experiments that high concentrations of a buffering agent (e.g. Tris buffer, acetate buffer and citric acid) can affect growth in a pH dependent manner.

Especially, low pH buffering (pH < 5) of yeast media is challenged by the fact that the only cheaply available buffering agents are weak organic acids like acetate or citric acid. Acetate is known to be toxic to cells at pH values below its dissociation constant (p*K*a = 4.76) [16, 17]. The then predominantly protonated form can enter cells via passive diffusion, leading to acidification of the cytosol and thus forcing the cell to invest energy (ATP) into maintaining a neutral cytosol [18]. Also citric acid is known to affect growth at high concentrations: with three carboxyl groups, citrate is a chelating compound and complexes several trace elements needed for growth [19]. Another buffering option is potassium hydrogen phthalate (KHP) but its high price makes it unsuitable for media development [19].

Most studies that require to work with a precisely adjusted low media pH use citric acid as part of the citrate phosphate buffer system (CPB) also known as McIlvaine buffer [20]. CPB is practical for media development for two reasons: first, the tri-protic nature of citric acid and disodium phosphate with three pKa values each, allows to buffer across a wide range of pH values (pH 2.2 to pH 8.0). Second, CPB allows to set a desired pH using defined volumes of citric acid and Na_2_HPO_4_ stock solutions. This is practical as no pH adjustment after media mixing is necessary and various pH values can be rapidly mixed from the same stock solutions. Unfortunately, often no exact protocols for media preparation or data on pH stability and growth in different media types are provided [21–25].

Despite the importance of pH maintenance for the development of bioprocesses in shaken batch cultures or microtiter plates there are to our knowledge yet no systematic studies that compare or develop a buffered media system that uses necessary but sufficient concentrations of a buffer agent in order to reliably maintain a desired culture pH across a wide pH range without affecting growth. As such, the goal of our study was to develop such a buffered growth media for *S. cerevisiae* that is also applicable to other biotechnologically relevant yeast; herein tested for *K. lactis*. Because of the practicality of the CPB we decided to make that the basis of this buffered media system.

## Results

### The initial media pH and the pH-change over biomass production in unbuffered SD media can be modulated with the nitrogen source

**Fig. 1:**
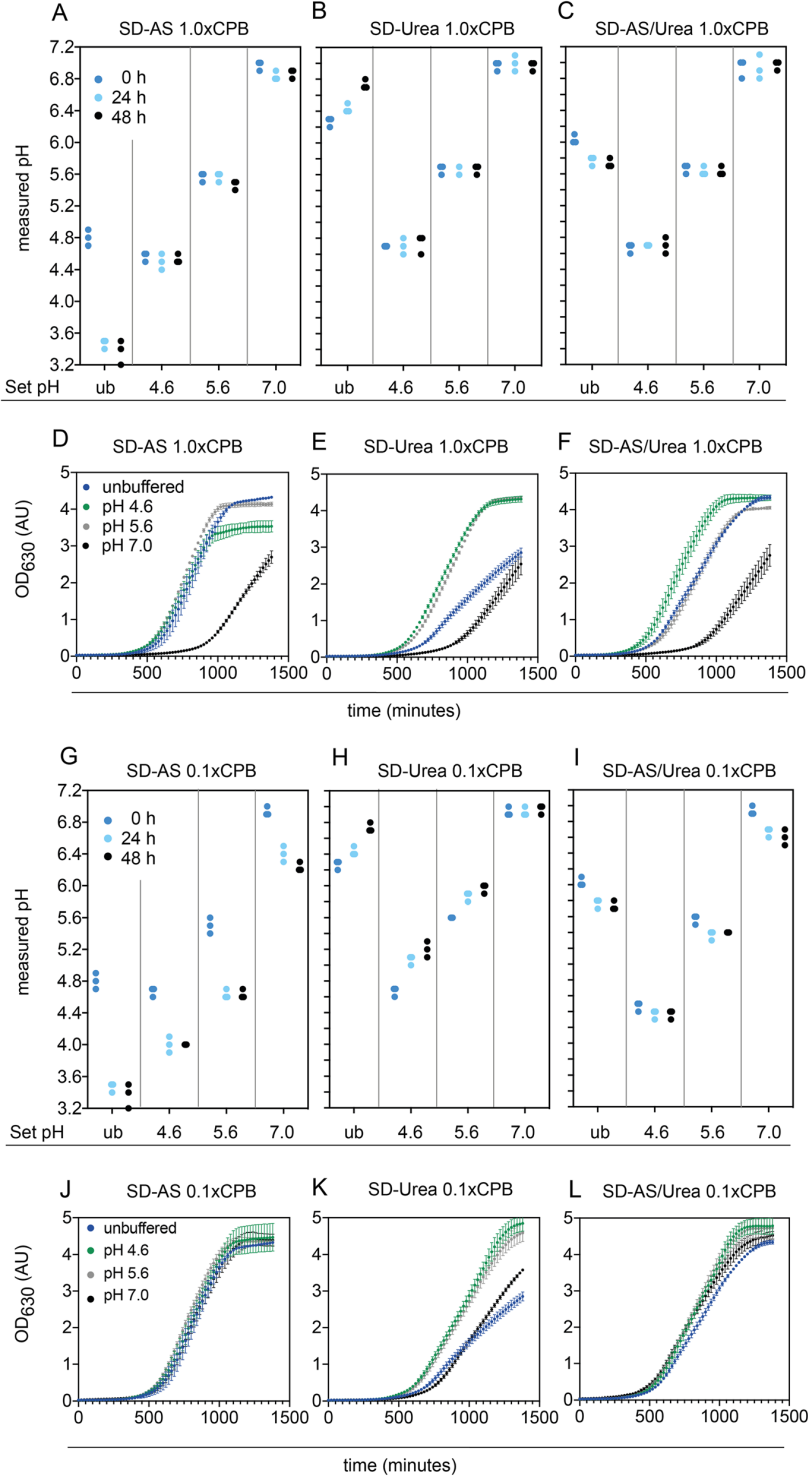
pH stability and growth under unbuffered conditions or when using 1.0x and 0.1x CPB across different media types and pH values. **a-c**: pH stability over 48 h in unbuffered (ub) media and across three pH values when using 1.0x CPB in SD-AS media **a**, SD-Urea media **b** and SD-AS/Urea media **c**. Individual values of triplicates are plotted. **d-f**: Growth across unbuffered (ub) media and when using 1.0x CPB in SD-AS media **d**, SD-Urea media **e** and SD-AS/Urea media **f**. Error bars represent the standard deviation of triplicates. **g-i**: pH stability in unbuffered (ub) media and across three pH values when using 0.1x CPB in SD-AS media **g**, SD-Urea media **h** and SD-AS/Urea media **i**. Individual values of triplicates are plotted. **j-l**: Growth across unbuffered media and when using 1.0x CPB in SD-AS media **j**, SD-Urea media **h** and SD-AS/Urea media **l**. Error bars represent the standard deviation of triplicates

The widely used synthetic complete drop-out (SD) media that uses 2% glucose as carbon source and ammonium sulfate (AS, (NH_4_)_2_SO_4_) as sole nitrogen source acidifies during biomass production [4, 26, 10]. It was discussed that this acidification is due to the stoichiometric release of protons during the consumption of ammonium salts [4], others studies report the accumulation of organic acids in the culture medium [10]. It has also been shown that acidification can be circumvented by replacing ammonium sulfate with urea as nitrogen source, or by decreasing the glucose concentration [4, 26]. As decreasing the glucose concentrations changes biomass yield – an important factor for biotechnological processes – we decided to not vary the carbon source but rather use the nitrogen source for pH modulation. We first re-tested the change in pH over biomass production in normal SD media with ammonium sulfate (herein called SD-AS media, 5 g/L AS), and in SD media with the ammonium sulfate replaced by urea (SD-Urea media, 5 g/L urea). Throughout the study we used the *S. cerevisiae* strain BY4741 as test case. As expected, the normal SD-AS media showed an average starting pH of 4.8 and was acidified during biomass production over 48 h to an average pH of 3.4 (Fig. 1 A, left panel). SD-Urea media showed a starting pH of 6.3 and slightly alkalized during biomass production to a pH of 6.7 (Fig. 1 B, left panel). Alkalization of urea media was also observed in a previous study with *K. lactis* [4]. As the use of ammonium sulfate leads to acidification of the media and the use of urea to alkalization, we were interested if a mixture of both nitrogen sources could modulate the pH change during biomass production in unbuffered media. We herein tested a 1:1 ratio (w/w) and the corresponding media will be referred to as SD-AS/Urea media (2.5 g/L AS and 2.5 g/L urea). Indeed, biomass production only slightly acidified this media, with its pH dropping from 6.1 to 5.7 over the course of 48 h (Fig. 1 C, left panel).

### The pH stability of CP-buffered media is enhanced when using a mixture of ammonium sulfate and urea as nitrogen source

Next, we tested if the pH of each media type could be effectively buffered with CPB and we chose pH 4.6, pH 5.6 and pH 7.0 as our test values. This pH range is most important for our work with small molecule antifungals and proteinaceous killer toxins which all show pH dependent functionalities. Further, media buffered at pH 7.0 is relevant for growth selections with commonly used antibiotic markers [7]. The herein used 1.0x CPB recipe adjusts the media pH with defined volumes of sterile stock solutions of 1 M citric acid and 1 M Na_2_HPO_4_ (Supplementary Table S1). In order to allow for simple media preparation from sufficiently concentrated stock solutions, we adjusted the stock concentrations and the required volumes from the original buffer table that uses 0.2 M Na_2_HPO_4_ and 0.1 M citric acid [20].

The final concentration of citric acid and Na_2_HPO_4_ in the media depends on the desired pH. For the tested pH values, they ranged between 17.6 mM and 53.2 mM citric acid and 93.5 mM and 164.7 mM Na_2_HPO_4_. In order to determine differences in the buffering capacity as well as to determine the minimal CPB concentration required to achieve pH stability, we tested pH stability in media buffered with 0.1x CPB (10 times less of the above indicated concentrations). Experiments were performed in 50 ml liquid culture in 125 ml shake flasks. The pH was measured by taking 7 ml samples before inoculation (0 h) and after 24 h and 48 h of growth. Fig. 1 A-C shows that 1.0x CPB allowed to adjust and maintain the media pH at its intended value for all media types. In contrast to 1.0x CPB, 0.1x CPB was not sufficient to maintain the pH of SD-AS media and SD-Urea media. The media either acidified or alkalized (Fig. 1 G and H). Only in the case of SD-AS/Urea media the pH stayed relatively stable (pH changes: from average pH 4.5 to 4.4, from pH 5.6 to 5.4, from pH 6.9 to 6.6) (Fig. 1 I**)**.

### CPB affects growth in a concentration dependent manner

Besides pH stability we were also interested in potential growth defects caused by the buffering agents or the pH. Therefore, we measured full growth curves for each media type and each pH compared to unbuffered media. Fig. 1 D-F shows that 1.0x CPB caused pH-dependent growth effects across all media types when compared to the unbuffered media control. For example, across all media types, buffering to pH 7.0 showed long lag phases and decreased growth rates (Supplementary Fig. S1). On the contrary, buffering to pH 4.6 and 5.6 showed enhanced growth rates in SD-Urea media, and buffering to pH 4.6 showed higher growth rates in SD-AS/Urea media (Supplementary Fig. S1). Buffering with 0.1x CPB allowed for normal growth in SD-AS and SD-AS/Urea media across all pH values. Noteworthy, while growth rates in unbuffered SD-AS and SD-AS/Urea were comparable, growth in SD-Urea media showed a much longer lag phase and slower growth rate (Supplementary Fig. S1B). As the SD-AS/Urea showed better pH performance we were – for the purpose of this study – not further interested in optimizing growth performance in SD-Urea media; thus, we excluded the SD-Urea media from further experiments.

In summary, at this point, 0.1x CP-buffered SD-AS/Urea media performed the best for our purpose. This media type allowed for normal growth across all tested pH values as well as it maintained all pH levels reasonably stable across biomass production (average pH changes: from pH 4.5 to 4.4, from pH 5.5 to 5.4 and from pH 6.9 to 6.4).

**Fig. 2:**
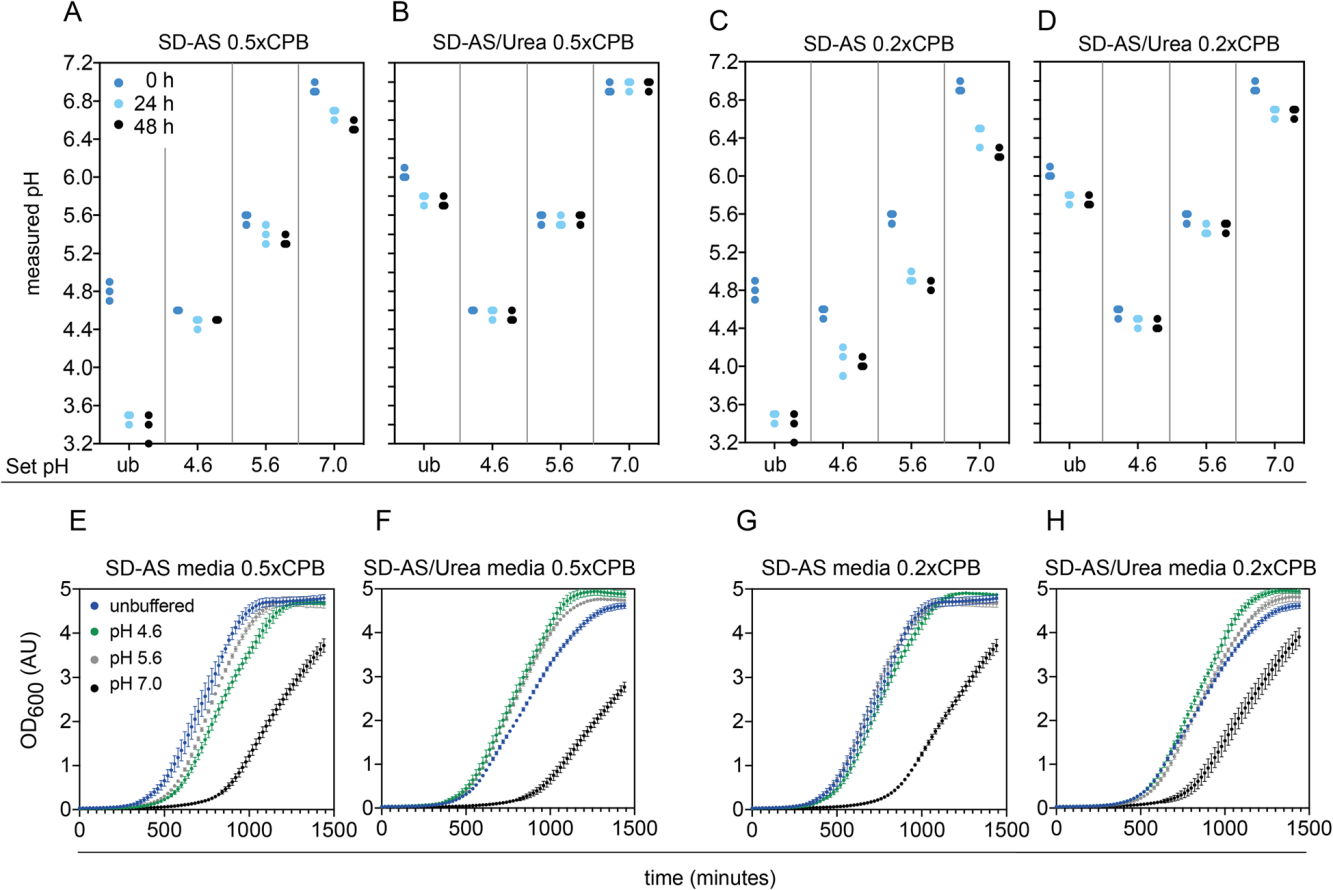
pH stability and growth in SD-AS and SD-AS/Urea media at 0.5xand 0.2x CPB. **a-b**: pH stability over 48 h in unbuffered (ub) media and across three pH values when using 0.5x CPB in SD-AS media **a** and SD-AS/Urea media **b**. **c-d**: pH stability in unbuffered (ub) media and across three pH values when using 0.2x CPB in SD-AS media **c** or SD-AS/Urea media **d**. Individual values of triplicate experiments are plotted. **e-f**: Growth across unbuffered SD-AS media **e** and SD-AS/Urea media **f** or when using 0.5x CPB. **g-h**: Growth across unbuffered SD-AS media **g** and SD-AS/Urea **h** media or when using 0.2x CPB. Error bars represent the standard deviation of triplicates

Still, as the pH maintenance at 0.1x CPB was not perfect we further tested if pH stability could be enhanced in 0.2x and 0.5x CPB, while maintaining stable growth. In terms of growth, both concentrations caused the same long lag phase for pH 7.0, but the growth phenotypes at pH 4.6 and 5.6 were not altered for both media types **(**Fig. 2 E-H and Supplementary Fig. S2). In terms of pH change, the pH only changed slightly when SD-AS media was buffered with 0.5x CPB, but significantly in the 0.2x CPB case (Fig. 2 A and C). In SD-AS/Urea media the pH remained stable for 0.5x CPB and showed slight changes when buffered with 0.2x CPB (Fig. 2 B and D). Those changes were comparable to the 0.1x CPB case (Fig. 1 I).

In summary, buffering with 0.5x CPB appeared to be a suitable option for low pH buffering in either SD-AS media and SD-AS/Urea media (as tested for pH 4.6 and pH 5.6).

### Tris/HCl does not effectively buffer the tested media types at neutral pH

**Fig. 3:**
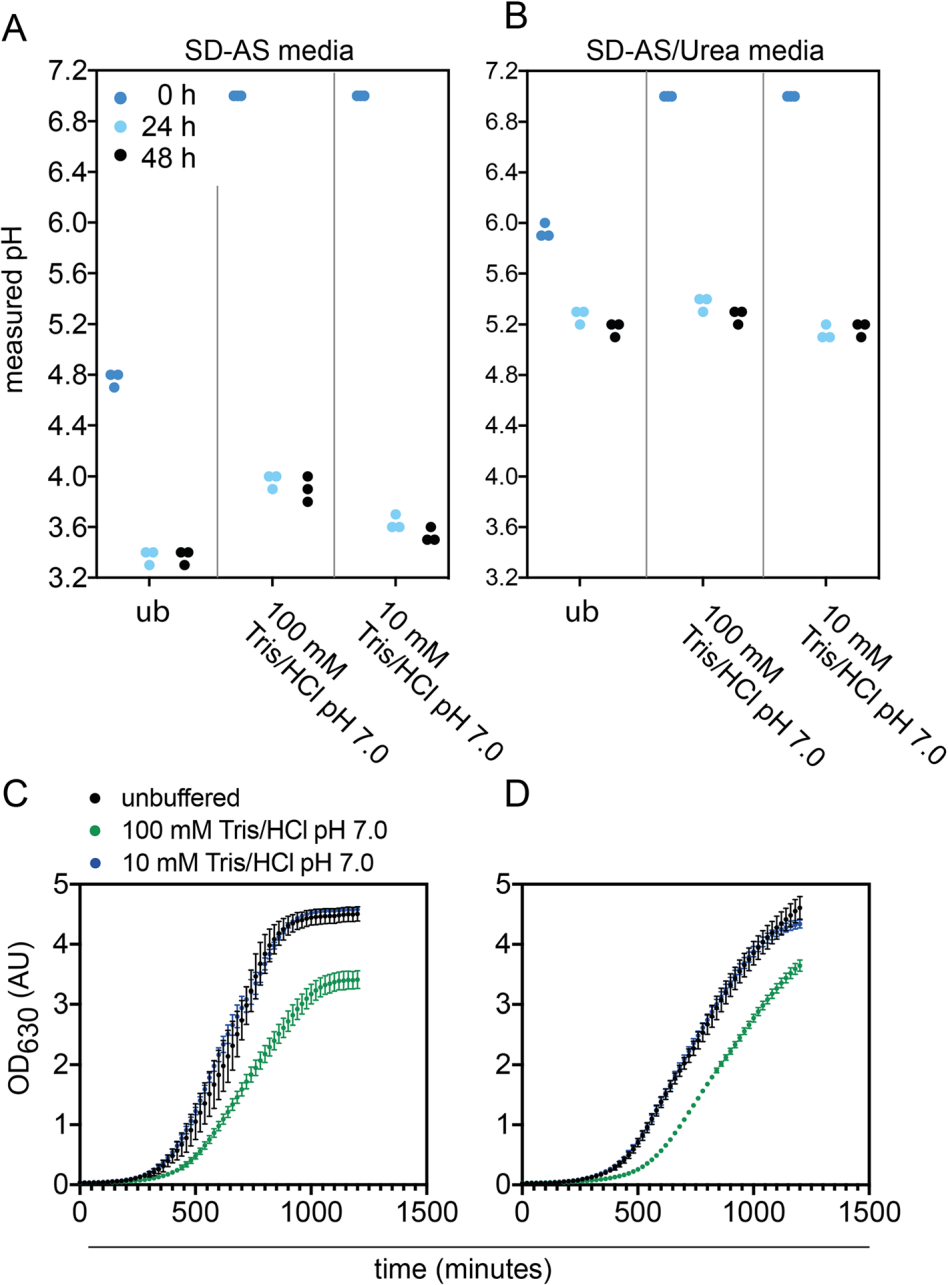
pH stability and growth in Tris-buffered SD-AS and SD-AS/Urea media. **a-b:** pH stability over 48 h in unbuffered (ub) SD-AS **a** and SD-AS/Urea media **b** and when buffered with 100 mM or 10 mM Tris/HCl pH 7.0. Individual values of triplicate experiments are plotted. **c-d**: Growth across unbuffered SD-AS media **c** and SD-AS/Urea media **b** or when buffered with 100 mM or 10 mM Tris/HCl pH 7.0. Error bars represent the standard deviation of triplicates

So far, our results have shown that buffering at neutral pH is the most challenging. CPB concentrations above 0.1x caused strong growth effects at pH 7.0 and were not enough to fully stabilize a neutral pH over biomass production. Several buffer agents with buffering capacities around neutral pH are available and widely used in biochemistry, molecular biology and microbiology. For example, Tris buffer has been used before to buffer yeast media a pH 7.0 [27]. Therefore, we tested if 10 mM or 100 mM Tris/HCl adjusted to pH 7.0 would be better suited for buffering at pH 7.0. We prepared media by supplementing SD-AS and SD-AS/Urea with appropriate amounts of a 500 mM stock solution. First, we noticed that simply adding Tris buffer did not suffice to adjust the media pH to 7.0. It rather yielded a starting pH of 6.3 and 6.7 for SD-AS and SD-AS/Urea media respectively buffered with 100 mM Tris/HCl pH 7.0, and 6.1 and 6.3 for 10 mM Tris/HCl pH 7.0. We then adjusted the media to pH 7.0 with 5 M NaOH and followed the pH changes over 48 h biomass production. We observed that even 100 mM Tris buffer was not sufficient to maintain the pH of either media. The pH dropped from pH 7.0 to 3.9 over 48 h of biomass production in SD-AS media (Fig. 3 A) and from pH 7.0 to 5.3 for SD-AS/Urea media (Fig. 3 B). Full growth curves demonstrate that 100 mM Tris buffer slowed growth, whereas 10 mM did not affect growth (Fig. 3 C and D).

### The best performing buffered media system is also suitable for other yeasts

**Fig. 4:**
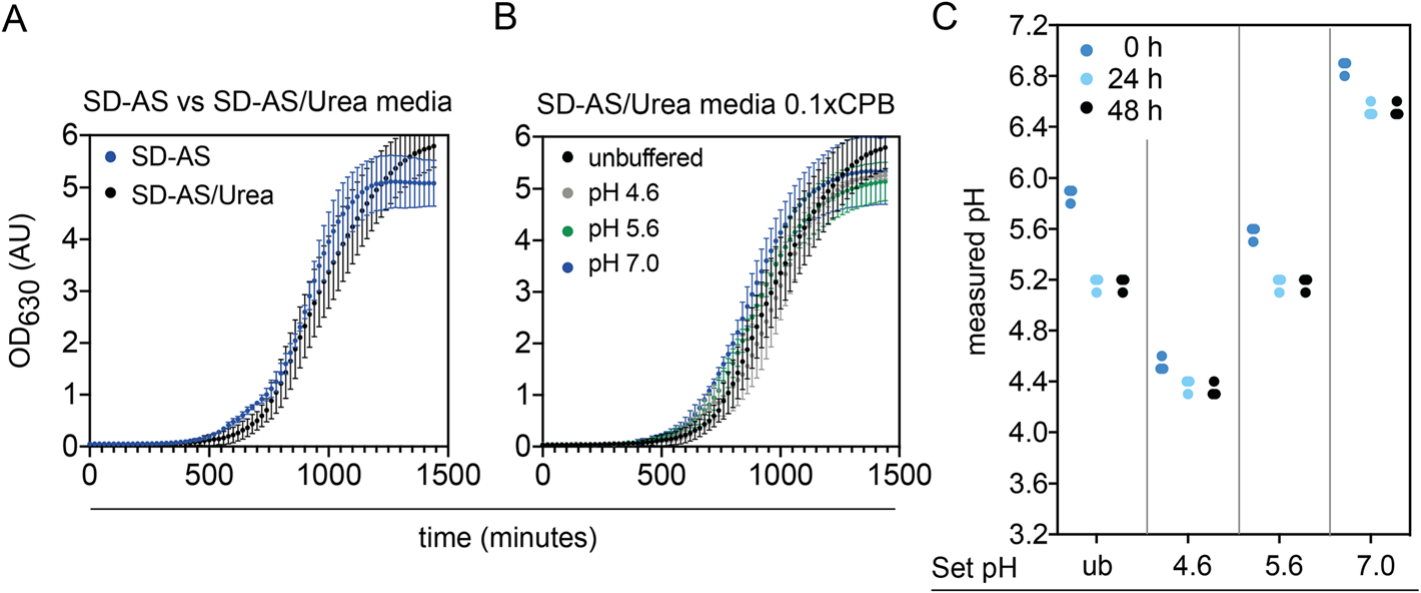
pH stability and growth in 0.1x CPB SD-AS/Urea media for *K. lactis*. **a**: Overlay of growth curves of *K. lactis* in unbuffered SD-AS and SD-AS/Urea media. **b**: Growth of *K. lactis* in unbuffered SD-AS/Urea media and when 0.1x CP-buffered at the indicated pH values. **c**: pH stability over 48 h in unbuffered (ub) SD-AS/Urea media and when buffered with 0.1xCPB. Individual values of triplicate experiments are plotted

Finally, we tested if our best performing buffered media system (SD-AS/Urea, 0.1x CPB) could also be used for buffering shake flask experiments of other biotechnologically relevant yeasts – such as *K. lactis* – across a wide pH range. We first verified that *K. lactis* shows no changed growth phenotype in media with part of the ammonium sulfate replaced by urea (Fig. 4 A). We further did not observe any altered growth phenotypes in *K. lactis* in either medium across all pH values (Fig. 4 B). Similar as for *S. cerevisiae*, the pH was reasonably stable over biomass production (average pH changes: from pH 4.5 to 4.3, from pH 5.6 to 5.2 and from pH 6.9 to 6.5).

## Discussion

Here we show that the effective buffering of yeast batch culture media is not straight-forward and requires some considerations in order to maintain a set pH and robust growth during biomass production. Media acidification by yeast has been observed before [4] as well as in this study. An important implication of this media acidification for existing and ongoing research is that a set culture pH (even when buffered) may change over biomass production, and that these pH changes may compromise interpretations of results in case the culture pH is an important experimental parameter. As such we recommend measuring the culture pH during or after growth in case of such pH-sensitive experiments.

We demonstrate that the media type with both urea and ammonium sulfate as nitrogen sources, buffered with a citrate-phosphate buffer (SD-AS/Urea), has the best buffering capacity without affecting growth. This buffering system allows for accurate and stable pH maintenance over biomass production.

Concerning pH accuracy, we observed some technical details of importance when preparing the SD-AS/Urea media: First, when using newly prepared media stock solutions there can be a batch to batch variability in initial and final pH. For example, our first batch of unbuffered SD-AS/Urea media showed a starting pH of 6.1 and dropped to pH 5.7 over biomass production, while our second batch showed a starting pH of 6.0 and dropped to pH 5.2 (Fig. 1 C, left panel and Fig. 4 C, left panel). This might be due to slight variations in the ammonium sulfate and urea concentrations in the media. The buffering capacity in the presence of 0.1x CPB was not affected (Fig. 4 C). Second, slight differences can also arise when independently setting media pH for different solutions. In our study this can be observed in the starting pH of the triplicate 1.0x CPB experiments (Fig. 1 a-c). In our experience, accurately pipetting the CPB solutions allows to adjust the media pH to an accuracy of plus minus 0.1 pH values (in rare cases 0.2 pH values). For simplicity, we did not further adjust the pH to the intended pH in those cases, but this is easily done by titrating in small amounts of one of the sterile stock solutions.

Buffering at pH 7.0 proved to be the most challenging, as CPB concentrations required for pH maintenance caused a long lag phase and a lower final OD_630_ during growth. Tris buffer has been used before to buffer yeast media at pH 7.0 [27], but 100 mM Tris buffer (pH 7.0) was not effective in our study to maintain neutral media pH in either tested medium. pH 7.0 falls at the lower end of the buffering capacity of the Tris buffer (range pH 7.0–9.0), and the decrease in pH was substantial. The observation that usual concentrations of buffers are not effective in buffering yeast media was made before: [8] For example, 10 to 30 mM bicine(N,N-Bis (2-hydroxyethylglycine))-TEA (triethanolamine) was not enough to buffer complex yeast media (YPD) at pH 8.0 or the same amounts of morpholino ethane sulfonic acid (MES)-TEA were not enough to buffer yeast media at pH 6.0.

Here, we did not test buffering systems with broader buffer capacity around pH 7.0, such as Bis-Tris (5.8–7.2). Bis-Tris has been used in mammalian cell cultures [28], but has not – to the best of our knowledge - been reported for use in yeast cultures. We did not test further buffers, as we found that 0.1x CPB in our SD-AS/Urea media worked reasonably well at maintaining pH 7.0. Still, it can be worthwhile testing other buffer systems if highly accurate pH maintenance around pH 7.0 is required.

## Conclusion

Using the herein presented cross-comparison we extract the following recommendations for buffering batch culture media for yeast:

1. Buffering at low pH < 5 (herein tested for pH 4.6): For studies that require a fixed low pH and seek to maintain the use of the common synthetic drop-out media (SD media) with ammonium sulfate, reasonable buffering with no changes in growth can be achieved by using SD-AS 0.5x CPB media (average pH change from 4.6 to 4.4 over 48 h). For studies that are flexible about the nitrogen source, we recommend to use SD-AS/Urea 0.5x CPB, as the buffering capacity is higher (no change in pH). We recommend to re-test exact media conditions for pH values other than 4.6 (e.g. using the presented work flow).
2. Buffering at pH 5 to 6 (herein tested for pH 5.6): We recommend the same as for point 1.
3. Buffering at neutral pH (herein tested for pH 7.0): We recommend to use SD-AS/Urea 0.1x CPB which did not cause growth defects and kept the pH reasonably stable.
4. Comparison across pH: For studies that seek to compare a specific trait across a wide range of pH values we recommend to use SD-AS/Urea 0.1x CPB.

## Methods

### Materials

Media and buffer components were obtained from BD Bioscience (Franklin Lakes, NJ, USA) and Sigma Aldrich (Darmstadt, Germany). Specifically yeast nitrogen base without amino acids and ammonium sulfate was obtained from Sigma and yeast nitrogen base without amino acids but with ammonium sulfate was obtained from BD Bioscience.

Sterile, transparent round-bottom microtiter plates were obtained from Corning (Corning Inc.).

### Strains

*Saccharomyces cerevisiae* BY4741 [29] and *Kluyveromyces lactis* NRRL-Y-1140 (derived from ATCC) were used for pH measurements and growth experiments.

### Media preparation

All media recipes are provided in Supplementary Tables S2, S3, S4, S5.

### pH measurements

Cells were grown overnight in 8 ml unbuffered SD-AS, SD-Urea or SD-AS/Urea medium. Triplicate shake flasks with 50 ml of each buffered media type were prepared and the initial pH (zero-hour time point) of a 7 ml sample was measured using a pH meter from Hanna Instruments 8520 (Nieuwegein, Netherlands). Each flask was then inoculated from the appropriate overnight culture (e.g. all buffered SD-AS media were inoculated from the unbuffered SD-AS medium culture) with a starting OD_630_ of 0.1. After 24 h and 48 h a 7 ml sample was taken, cells were separated by centrifugation for 30 min at 4000 rpm in a Beckman Coulter Allegra X-15R centrifuge, and pH of the supernatant was measured after transfer into a fresh tube.

### Growth measurements

Growth curves across media types were recorded in sterile, transparent round-bottom 96-well plates using 200 μl total culture volume, cultured at 30 °C in a SynergyMx plate reader (high orbital shaking). Cells were seeded at an OD_630_ of approximately 0.03 and culture turbidity (OD_630_) was recorded every 20 min for 20 to 24 h. Since the later optical density values were outside the linear range of the photodetector, all optical density values were first corrected using the following formula to calculate true optical density values:

Eq. 1 $$ {OD}_{true}=\frac{k\bullet {OD}_{meas}}{OD_{sat}-{OD}_{meas}} $$

where *OD*_*meas*_ is the measured optical density, *OD*_*sat*_ is the saturation value of the photodetector (2.568 for our instrument, as experimentally determined) and *k* is the true optical density at which the detector reaches half saturation of the measured optical density (2.075 for our instrument, as experimentally determined). Specific growth rates were determined by linear regression of the manually determined linear range of the Log2(OD_630_) versus time plots of each growth curve.

## Supplementary Information

**Additional file 1: Supplementary Table S1.** Citrate-Phosphate Buffer (CPB) Stock solutions. **Supplementary Table S2.** SD-AS media recipes across CPB concentrations. **Supplementary Table S3.** SD-Urea media recipes across CPB concentrations. **Supplementary Table S4.** SD-AS/Urea media recipes across CPB concentrations. **Supplementary Table S5.** 20x amino acid solution recipe. **Supplementary Figure S1.** Growth rates and final OD_630_ across media types and pH values when buffered with 1.0x and 0.1x CPB. **Supplementary Figure S2.** Growth rates and final OD_630_ across media types and pH values when buffered with 0.2x and 0.5x CPB.

## Data Availability

The datasets used and/or analysed during the current study are available from the corresponding author on reasonable request.
