# Supplementary material for: A buffered media system for yeast batch culture growth
Source: BMC Microbiol. 2021 Apr 23;21:127. doi: 10.1186/s12866-021-02191-5 (PMC8063419; doi:10.1186/s12866-021-02191-5)
Supplement: Supplementary file 1 — Additional file 1: Supplementary Table S1. Citrate-Phosphate Buffer (CPB) Stock solutions. Supplementary Table S2. SD-AS media recipes across CPB concentrations. Supplementary Table S3. SD-Urea media recipes across CPB concentrations. Supplementary Table S4. SD-AS/Urea media recipes across CPB concentrations. Supplementary Table S5. 20x amino acid solution recipe. Supplementary Figure S1. Growth rates and final OD630 across media types and pH values when buffered with 1.0x and 0.1x CPB. Supplementary Figure S2. Growth rates and final OD630 across media types and pH values when buffered with 0.2x and 0.5x CPB. [file 12866_2021_2191_MOESM1_ESM.docx]

**Supplementary Material**

Prins et al.

**A buffered media system for yeast batch culture growth**

Content:

**Supplementary Table S1:** Citrate-Phosphate Buffer (CPB) Stock solutions.

**Supplementary Table S2:** SD-AS media recipes across CPB concentrations.

**Supplementary Table S3:** SD-Urea media recipes across CPB concentrations.

**Supplementary Table S4:** SD-AS/Urea media recipes across CPB concentrations.

**Supplementary Table S5:** 20x amino acid solution recipe.

**Supplementary Figure S1:** Growth rates and final OD_630_ across media types and pH values when buffered with 1.0x and 0.1x CPB.

**Supplementary Figure S2:** Growth rates and final OD_630_ across media types and pH values when buffered with 0.2x and 0.5x CPB.

**Supplementary Table S1. Citrate-Phosphate Buffer (CPB) Stock solutions.** The solutions and volumes were adjusted from the original protocol^1^ such that volumes of 1 M sterile stock solutions Na_2_HPO_4_ and Citric Acid can be directly added to 100 ml (final volume) medium.

| **For 100 mL** | **1 M** **Na_2_HPO_4_** | **1 M** **Citric Acid** |
| --- | --- | --- |
| **Desired pH** | **ml** | **ml** |
| 2.2 | 0.400 | 9.800 |
| 2.4 | 1.240 | 9.380 |
| 2.6 | 2.180 | 8.910 |
| 2.8 | 3.170 | 8.415 |
| 3.0 | 4.110 | 7.945 |
| 3.2 | 4.940 | 7.530 |
| 3.4 | 5.700 | 7.150 |
| 3.6 | 6.440 | 6.780 |
| 3.8 | 7.100 | 6.450 |
| 4.0 | 7.710 | 6.145 |
| 4.2 | 8.280 | 5.860 |
| 4.4 | 8.820 | 5.590 |
| 4.6 | 9.350 | 5.325 |
| 4.8 | 9.860 | 5.070 |
| 5.0 | 10.300 | 4.850 |
| 5.2 | 10.720 | 4.640 |
| 5.4 | 11.150 | 4.425 |
| 5.6 | 11.600 | 4.200 |
| 5.8 | 12.090 | 3.955 |
| 6.0 | 12.630 | 3.685 |
| 6.2 | 13.220 | 3.390 |
| 6.4 | 13.850 | 3.075 |
| 6.6 | 14.550 | 2.725 |
| 6.8 | 15.450 | 2.275 |
| 7.0 | 16.470 | 1.765 |
| 7.2 | 17.390 | 1.305 |
| 7.4 | 18.170 | 0.915 |
| 7.6 | 18.730 | 0.635 |
| 7.8 | 19.150 | 0.425 |
| 8.0 | 19.450 | 0.275 |

**Supplementary Table S2.** SD-AS media recipes across CPB concentrations (1.0x to 0.1x).

| **For 100 ml of 2x media (in ml)** | **unbuffered** | **pH 4.6** | **pH 5.6** | **pH 7.0** |
| --- | --- | --- | --- | --- |
| 10x Yeast Nitrogen Base Without Amino Acids and Ammonium Sulfate (17g/L) | 0 | 0 | 0 | 0 |
| 10x Urea solution (50g/L) | 0 | 0 | 0 | 0 |
| 10x Yeast Nitrogen Base Without Amino Acids with Ammonium Sulfate (67g/L) | 20 | 20 | 20 | 20 |
| 40% Glucose | 10 | 10 | 10 | 10 |
| 20x Amino Acids^a^ | 10 | 10 | 10 | 10 |
| 1% Histidine | 0.4 | 0.4 | 0.4 | 0.4 |
| 1% Tryptophan | 0.4 | 0.4 | 0.4 | 0.4 |
| 0.2% Uracil | 2 | 2 | 2 | 2 |
| 1% Leucine | 1.8 | 1.8 | 1.8 | 1.8 |
| 1M Na_2_HPO_4_ | 0 | 1.0x: 18.700  0.5x: 9.350  0.2x: 3.740  0.1x: 1.870 | 1.0x: 23.200  0.5x: 11.600  0.2x: 4.640  0.1x: 2.320 | 1.0x: 32.940  0.5x: 16.470  0.2x: 6.580  0.1x: 3.290 |
| 1M Citric acid | 0 | 1.0x: 10.650  0.5x: 5.325  0.2x: 2.130  0.1x: 1.065 | 1.0x: 8.400  0.5x: 4.200  0.2x:1.680  0.1x: 0.840 | 1.0x: 3.530  0.5x: 1.765  0.2x: 0.706  0.1x: 0.353 |
| H_2_O | 55.4 | 1.0x: 26.050  0.5x: 4.073  0.2x: 49.650  0.1x: 52.465 | 1.0x: 23.800  0.5x: 3.600  0.2x: 49.080  0.1x: 52.240 | 1.0x: 18.930  0.5x: 37.165  0.2x: 48.106  0.1x: 51.753 |

^a^ see Supplementary Table S5

**Supplementary Table S3.** SD-Urea media recipes across CPB concentrations (1.0x and 0.1x)

| **For 100 ml 2x media (in ml)** | **unbuffered** | **pH 4.6** | **pH 5.6** | **pH 7** |
| --- | --- | --- | --- | --- |
| 10x Yeast Nitrogen Base Without Amino Acids and Ammonium Sulfate (17g/L) | 20 | 20 | 20 | 20 |
| 10x Urea (50 g/L) | 20 | 20 | 20 | 20 |
| 10x Yeast Nitrogen Base Without Amino Acids with Ammonium Sulfate (67g/L) | 0 | 0 | 0 | 0 |
| 40% Glucose | 10. | 10 | 10 | 10 |
| 20x Amino Acids^a^ | 10 | 10 | 10 | 10 |
| 1% Histidine | 0.4 | 0.4 | 0.4 | 0.4 |
| 1% Tryptophan | 0.4 | 0.4 | 0.4 | 0.4 |
| 0.2% Uracil | 2 | 2 | 2 | 2 |
| 1% Leucine | 1.8 | 1,8 | 1,8 | 1,8 |
| 1M Na_2_HPO_4_ | 0 | 1.0x: 18.700  0.1x: 1.8700 | 1.0x: 23.200  0.1x: 2.320 | 1.0x: 32.940  0.1x: 3.290 |
| 1M Citric Acid | 0 | 1.0x: 10.65  0.1x: 1.065 | 1.0x: 8.400  0.1x: 0.840 | 1.0x: 3.530  0.1x: 0.353 |
| H_2_O | 35.4 | 1.0x: 6.050  0.1x: 32.465 | 1.0x: 3.800  0.1x: 32.240 | 1.0x: 0.000  0.1x: 31.753 |

^a^ see Supplementary Table S5

**Supplementary Table S4. SD-AS media recipes across CPB concentrations (1.0x to 0.1x)**

| **For 100 ml 2x media (in ml)** | **unbuffered** | **pH 4.6** | **pH 5.5** | **pH 7** |
| --- | --- | --- | --- | --- |
| 10x 10x Yeast Nitrogen Base Without Amino Acids and Ammonium Sulfate (17g/L) | 10 | 10 | 10 | 10 |
| 10x Urea (50g/L) | 10 | 10 | 10 | 10 |
| 10x Yeast Nitrogen Base Without Amino Acids with Ammonium Sulfate (67g/L) | 10 | 10 | 10 | 10 |
| 40% Glucose | 10 | 10 | 10 | 10 |
| 20x Amino Acids^a^ | 10 | 10 | 10 | 10 |
| 1% Histidine | 0.4 | 0.4 | 0.4 | 0.4 |
| 1% Tryptophan | 0.4 | 0.4 | 0.4 | 0.4 |
| 0.2% Uracil | 2 | 2 | 2 | 2 |
| 1% Leucine | 1.8 | 1.8 | 1.8 | 1.8 |
| 1M Na_2_HPO_4_ | 0 | 1.0x: 18.700  0.5x: 9.350  0.2x: 3.740  0.1x: 1.870 | 1.0x: 23.200  0.5x: 11.600  0.2x: 4.640  0.1x: 2.320 | 1.0x: 32.940  0.5x: 16.470  0.2x: 6.580  0.1x: 3.290 |
| 1M Citric Acid | 0 | 1.0x: 10.650  0.5x: 5.3250  0.2x: 2.130  0.1x: 1.065 | 1.0x: 8.400  0.5x: 4.200  0.2x: 1.680  0.1x: 0.840 | 1.0x: 3.530  0.5x: 1.765  0.2x: 0.706  0.1x: 0.353 |
| H_2_O | 45.4 | 1.0x: 16.050  0.5x: 30.725  0.2x: 39.650  0.1x: 42.465 | 1.0x: 13.800  0.5x: 29.600  0.2x: 39.080  0.1x: 42.240 | 1.0x: 8.930  0.5x: 27.165  0.2x: 38.106  0.1x: 41.753 |

^a^ see Supplementary Table S5

**Supplementary Table S5.** Recipe for 500 ml sterile filtered 20x amino acids.

| **Compound** | **Amount (g) for making 500 ml 20x amino acids** |
| --- | --- |
| Adenine Sulfate | 0.2 |
| Arginine HCl | 0.2 |
| Aspartic Acid | 1 |
| Glutamic Acid | 1 |
| Isoleucine | 0.3 |
| Lysine HCl | 0.3 |
| Methionine | 0.2 |
| Phenylalanine | 0.5 |
| Serine | 4 |
| Threonine | 2 |
| Tyrosine | 0.3 |
| Valine | 1.5 |

**
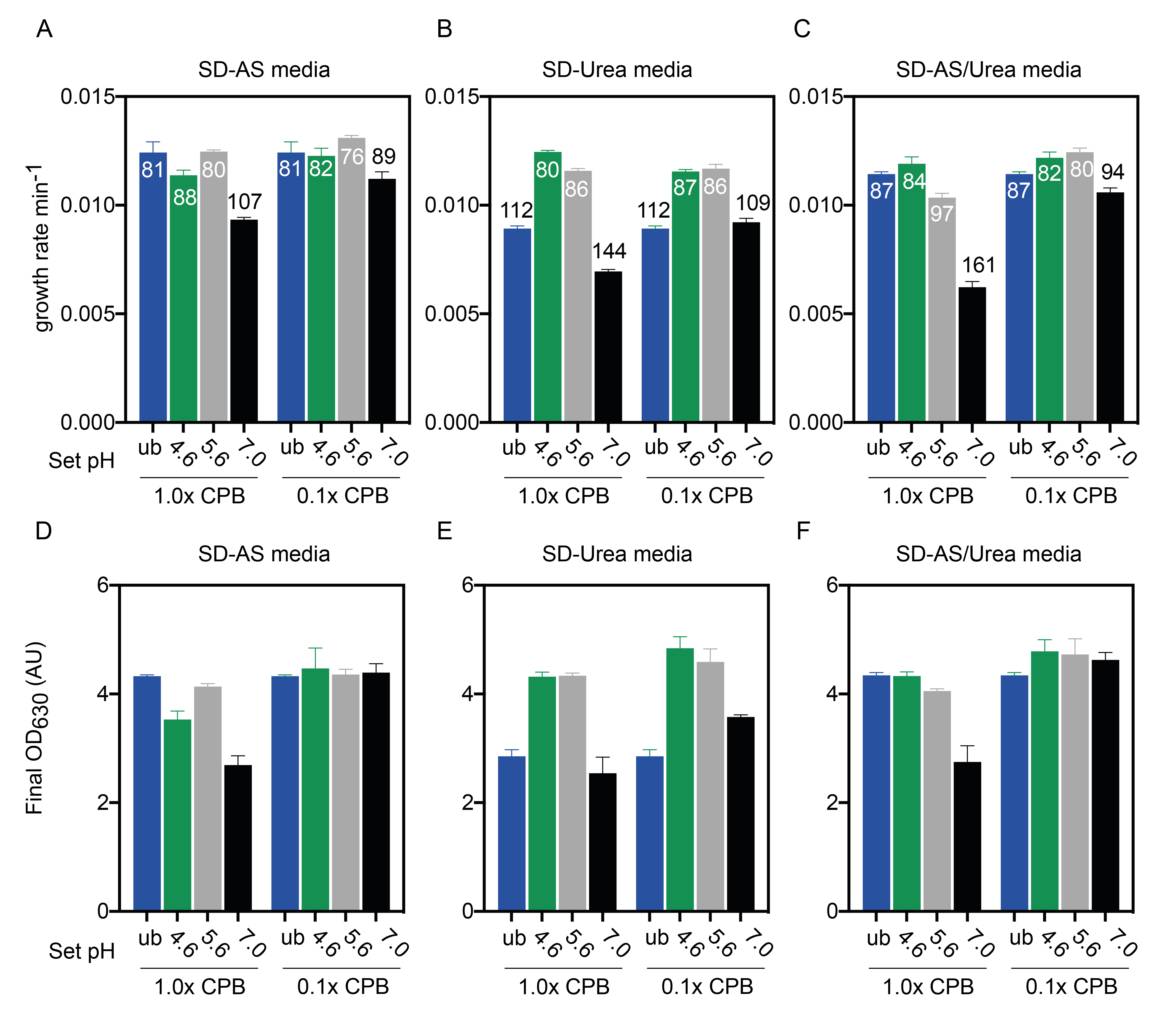
**

**Supplementary Figure S1. Growth rates and final OD_630_ across media types and pH values when buffered with 1.0x and 0.1x CPB. A-C**: Specific growth rates in unbuffered (ub) media and across three pH values when using 1.0x or 0.1x CPB in SD-AS media (A), SD-Urea media (B) and SD-AS/Urea media (C). The numbers above the bars indicate the average doubling time (min), calculated from the specific growth rate.

**D-F**: Final OD_630_ (after 23 hours of growth) in unbuffered (ub) media and across three pH values when using 1.0x or 0.1x CPB in SD-AS media (D), SD-Urea media (E) and SD-AS/Urea media (F). Error bars represent the standard deviation of triplicates.

**
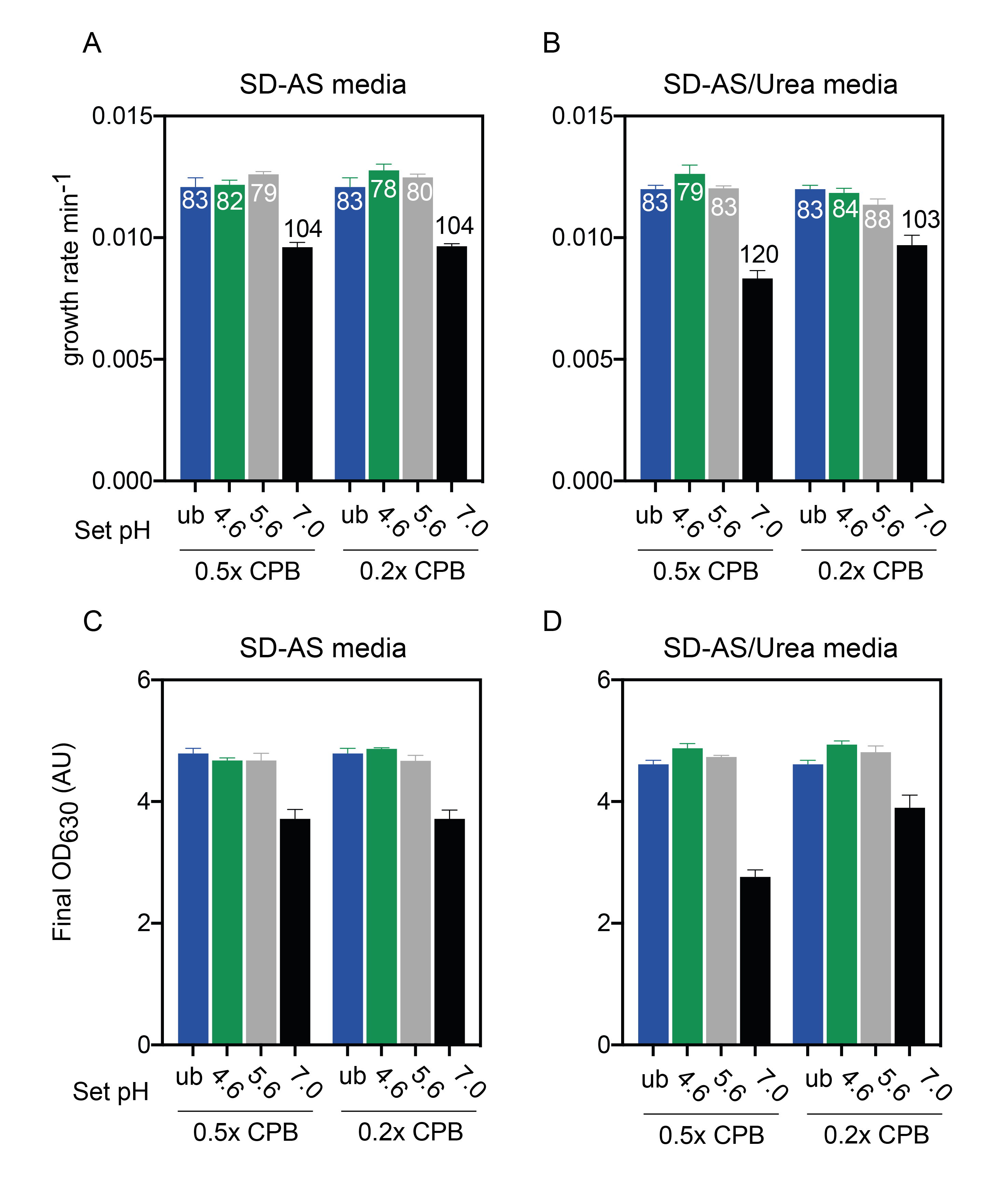
**

**Supplementary Figure S2. Growth rates and final OD_630_ across pH values when buffered with 0.5x and 0.2x CPB. A-B**: Specific growth rates in unbuffered (ub) media and across three pH values when using 0.5x or 0.2x CPB in SD-AS media (A) or SD-AS/Urea media (B). The numbers above the bars indicate the average doubling time (min), calculated from the specific growth rate.

**C-D**: Final OD_630_ (after 23 hours growth) in unbuffered (ub) media and across three pH values when using 0.5x or 0.2x CPB in SD-AS media (C) or SD-AS/Urea media (D). Error bars represent the standard deviation of triplicates.
